# Tobacco Smoking and Tuberculosis among Men Living with HIV in Johannesburg, South Africa: A Case-Control Study

**DOI:** 10.1371/journal.pone.0167133

**Published:** 2016-11-28

**Authors:** Liza Bronner Murrison, Neil Martinson, Rachael M. Moloney, Regina Msandiwa, Mondiwana Mashabela, Jonathan M. Samet, Jonathan E. Golub

**Affiliations:** 1 Department of Epidemiology, Johns Hopkins Bloomberg School of Public Health, Baltimore, Maryland, United States of America; 2 Center for Tuberculosis Research, Johns Hopkins School of Medicine, Baltimore, Maryland, United States of America; 3 Perinatal HIV Research Unit, MRC Soweto Matlosana Collaborating Centre for HIV/AIDS and TB, University of the Witwatersrand, Johannesburg, South Africa; 4 NRF/DST Center of Excellence for Biomedical TB Research, University of the Witwatersrand, Johannesburg, South Africa; 5 Department of Preventive Medicine, University of Southern California, Los Angeles, California, United States of America; Institute of Infectious Diseases and Molecular Medicine, SOUTH AFRICA

## Abstract

**Setting:**

Although there is ample evidence that smoking increases the risk of tuberculosis (TB), the magnitude of impact on TB risk among HIV-infected persons is poorly described. Given that a high proportion of patients with TB are co-infected with HIV in South Africa, the risks arising from the intersection of smoking, TB, and HIV/AIDS have key relevance for tobacco control policies.

**Objective:**

To evaluate the association of pulmonary tuberculosis (PTB) with current tobacco smoking among men with HIV in South Africa.

**Design:**

Case-control study of antiretroviral therapy naïve men with confirmed HIV-infection in Johannesburg. Cases had laboratory-confirmed PTB and controls had no evidence of active TB. Participants were interviewed to collect detailed smoking histories.

**Results:**

We enrolled 146 men diagnosed with PTB and 133 controls. Overall, 33% of participants were currently smoking, defined as smoking a cigarette within 2 months (34% cases vs. 32% controls, p = 0.27). Median CD4 count was lower (60 vs. 81 cells/mm3, P = 0.03) and median viral load was higher (173 vs. 67 copies/ul per thousand, P<0.001) among cases versus controls. In adjusted analyses, current smoking tripled the odds of PTB (aOR 3.2; 95%CI: 1.3–7.9, P = 0.01) and former smoking nearly doubled the odds of PTB (aOR 1.8; 95%CI 0.8–4.4, P = 0.18) compared to never smoking.

**Conclusions:**

Males with HIV that smoke are at greater odds for developing PTB than non-smokers. Extensive smoking cessation programs are needed to reduce odds of TB and promote health among adults living with HIV.

## Introduction

Although tuberculosis (TB) is a curable disease, 10.4 million incident TB cases occurred worldwide in 2015 [1]. Tobacco smoking is estimated to account for one-fifth of all TB cases globally [2], even in South Africa where the annual TB incidence rate (834 per 100,000) is among the highest in the world and HIV is the dominant risk factor for TB [1,3–5]. The HIV epidemic in South Africa is the largest in the world and includes 6.5 million adults living with HIV [6]. Among adults living with HIV, there is evidence that the prevalence of smoking is higher than that among adults not infected with HIV [7,8]. Additionally, patients with suspected and confirmed TB in South Africa have a higher prevalence of smoking compared to the one-third reported among the general population [5,9,10].

The 2014 U.S. Surgeon General’s Report implicates smoking as a cause of TB disease among those latently infected with *Mycobacterium tuberculosis (M*. *tb)* [11]. While there is ample evidence that smoking increases the risk of TB and risk of death from TB [10], data are scant on the extent to which smoking tobacco increases TB risk among people living with HIV. The large population of people living with HIV in South Africa represents a vulnerable population that may benefit from improved tobacco control. Given that a high proportion of patients with TB are co-infected with HIV in South Africa [1], the objective of this study was to investigate the association of smoking with active pulmonary TB (PTB) disease among men with HIV in South Africa.

## Methods

We conducted a case-control study of male adults with HIV comparing those with PTB to controls without TB recruited at the same institution in South Africa. From February 2009 to September 2010, male patients with PTB and control patients without TB were consecutively recruited from inpatient wards at Chris Hani Baragwanath Hospital (CHBH) in Soweto, South Africa; additionally, cases and controls were consecutively recruited from an outpatient HIV clinic on the campus of CHBH [12,13]. All eligible patients included in the research study were antiretroviral therapy (ART) naïve men, age 25 years or more with laboratory confirmed HIV. Cases were men with both HIV and PTB, confirmed by sputum smear microscopy or sputum culture, and/or a chest x-ray suggestive of TB in accordance with South African National TB Program Guidelines [14]. Cases with PTB were included independent of drug-sensitivity status. Controls were men with HIV with at least two prior visits in the past six months with stable weight (defined either as increasing weight or <2kg decrease) and not diagnosed with TB disease or any recorded symptoms suggestive of TB at the time of enrollment or in the six months prior to enrollment. We restricted enrollment to male patients because the prevalence of smoking is three times as high in men compared to women in South Africa [9,15].

All participants were interviewed using the same structured questionnaire to obtain details on their TB symptoms and duration, and a detailed cigarette smoking history including: ever/never smoked, age of smoking initiation, self-reported current smoking, number of cigarettes per day, recent quit attempts, and date of the last time that a cigarette was smoked; as well as alcohol use, demographic and socio-economic factors. We calculated pack years as: pack years = (number of cigarettes per day x number of years smoking)/20. We classified participants smoking status as current, former, and never based on the criteria used by the Centers for Disease Control and Prevention’s Global Adult Tobacco Survey [16]. We defined current smoking as self-reported smoking, including those who reported smoking within the 2-month period prior to enrollment. Our definition of current smoking includes periodicity beyond what is typically used (past 30 days or reporting use every day or some days) to include people who may have quit due to TB-related illness (e.g., cough), as TB symptoms are estimated to be present for 12 months prior to a TB diagnosis in South Africa [17,18]. We measured alcohol consumption as self-reported drinks per week. Participants were asked how many drinks per week they were currently drinking where one drink was defined as: one quart = 2 drinks, one tot of hard liquor = 1 drink, one glass of wine = 1 drink, one can of beer = 1 drink. We examined alcohol consumption classified by individuals drinking 0 (no), 1–14 (low), and ≥15 (heavy) drinks per week using the National Institute on Alcohol Abuse and Alcoholism definitions [19]. For participants who were missing data for monthly household income, we used their reported monthly personal income when available (n = 5).

### Statistical Analysis

We assessed differences in proportion between cases with PTB and controls for socio-demographic and smoking exposure indicators using Pearson's chi-square test for categorical variables and the nonparametric Mann-Whitney rank-sum test for continuous indicators. To evaluate differences in proportion between individuals reporting current, former, and never smoking, we used Pearson's chi-square test for categorical variables and the nonparametric Kruskal-Wallis test for continuous indicators. We explored predictors of PTB in univariable and multivariable logistic regression models comparing participants reporting current smoking and former smoking to those never smoking. Variables included in the final multivariable logistic regression model were those specified a priori and those that were significantly associated at the univariable level.

### Ethics

This study was approved by the Institutional Review Boards at Johns Hopkins School of Medicine in Baltimore, MD and the University of Witwatersrand in Johannesburg, South Africa. Written informed consent was obtained from all participants prior to being interviewed for the study.

## Results

### Socio-demographic and clinical characteristics

We interviewed 279 men with HIV for this study; 146 cases with PTB and 133 controls not diagnosed with TB. Median participant age was 38 years [IQR: 33–46] (Table 1). Cases were more likely to have education at the fifth grade level or lower (18% vs. 7%), be unemployed (60% vs. 44%), and have lower median monthly household income (ZAR 775 vs. 1700; approximately US$56 vs. $123) compared to controls. Median BMI was relatively high for both cases and controls, while median CD4 count was lower among cases and their median viral load was higher compared to controls. The median duration of HIV infection was shorter among cases compared to controls (38 days vs. 78 days).

**Table 1:** Socio-demographic and smoking history characteristics of controls without and cases with pulmonary TB in Soweto, Johannesburg, South Africa (n = 279).

	Controls (n = 133)	Cases (n = 146)		Unadjusted OR	
Characteristic°	n (%)	n (%)	*P*‡	(95% CI)*	*P*
Age (years), (n = 278)					
Median [IQR]	38 [33–45]	38 [33–46]	0.94	1.00 (0.98–1.03)	0.93
Education, (n = 272)					
5th grade or lower	9 (7)	26 (18)	**0.02**	Ref	
6th grade to 11th grade	87 (68)	92 (64)		**0.37 (0.16–0.83)**	**0.02**
12th grade or higher	32 (25)	26 (18)		**0.28 (0.11–0.70)**	**<0.01**
Employed, (n = 261)	69 (56)	55 (40)	**<0.01**	Ref	
Unemployed	54 (44)	83 (60)		**1.93 (1.18–3.16)**	**<0.01**
Monthly household income, (n = 245)†					
0–999 ZAR	53 (46)	80 (62)	**0.05**	Ref	
1000–4999 ZAR	47 (41)	39 (30)		**0.55 (0.32–0.95)**	**0.03**
≥5000 ZAR	15 (13)	11 (8)		**0.49 (0.21–1.14)**	**0.10**
Electricity to heat house, (n = 265)	104 (82)	100 (72)	0.07	0.58 (0.32–1.05)	0.07
No electricity to heat house	23 (18)	38 (28)		Ref	
BMI (kg/m2), (n = 239)					
Median [IQR]	23 [20–27]	23 [21–26]	0.97	0.99 (0.93–1.04)	0.60
CD4 (cells/mm3), (n = 237)					
Median [IQR]	81 [31–174]	60 [27–98]	**0.03**	1.00 (0.99–1.00)	0.08
Viral load (copies/ul, thousands), (n = 242)					
Median [IQR]^φ^	67 [21–268]	173 [56–500]	**<0.001**	**1.00 (1.00–1.00)**	**<0.001**
Duration of HIV Infection (days), (n = 208)					
Median [IQR]	78 [27–352]	38 [22–87]	**0.02**	1.00 (1.00–1.00)	0.15
Previous TB (n = 260)	15 (12)	18 (13)	0.82	1.09 (0.52–2.27)	0.82
No previous TB disease	108 (88)	119 (87)		Ref	
Current smoking (n = 267)**	43 (32)	49 (34)	0.27	1.43 (0.77–2.68)	0.26
Former smoking	51 (39)	66 (45)		1.63 (0.90–2.97)	0.11
Never smoking	39 (29)	31 (21)		Ref	
Cigarettes per day, (n = 168)					
Median [IQR]	2 [0–5]	3 [0–6]	0.20	1.04 (0.99–1.08)	0.10
Years smoking, (n = 188)					
Median [IQR]	20 [14–26]	22 [15–29]	0.27	1.02 (0.99–1.05)	0.29
Pack years, (n = 160)					
Median [IQR]	0.2 [0–4]	3 [0–7]	0.10	1.05 (1.00–1.10)	0.06
Alcoholic drinks/week, (n = 279)					
0 drinks/week	22 (17)	24 (16)	0.94	Ref	
1–14 drinks/week	92 (69)	99 (68)		0.99 (0.52–1.88)	0.98
≥ 15 drinks/week	19 (14)	23 (16)		1.11 (0.48–2.58)	0.81

°Total number of participants with data available listed in parentheses.

^‡^Pearson's chi-squared (or Fisher's exact) test for categorical variables comparing cases and controls; Wilcoxon test for continuous variables.

*Unadjusted odds ratio comparing cases and controls (95% confidence intervals).

^†^Participants were classified as having 0–999 ZAR monthly household income if income was unknown ad unemployed.

^ϕ^Numbers in table have been rounded to two decimal places. The odds ratio (95% CI) is: viral load 1.003 (1.001–1.004); HIV duration 0.999 (0.999–1.000).

**Last cigarette smoked relative to study interview date.

In univariate analyses, unemployment was associated with a 93% increase in the odds of PTB (OR 1.93, 95%CI: 1.18–3.16) (Table 1). The odds of PTB were lower among those who had higher than a fifth grade education, those with a monthly household income of ZAR 1000–4999 (US$72–360) or more, and those with electricity to heat their house.

### Smoking history

Overall, current smoking of a cigarette within 2 months of the interview date was reported by 33% (95%CI: 27–39%) of participants, while 42% (95%CI: 36–48%) reported former smoking history and 25% (95%CI: 20–30%) reported never smoking in their lifetime (Table 1). A greater proportion of cases reported ever smoking compared with controls (79% vs. 71%, P = 0.12). The median number of pack years smoking was higher among cases than controls (3 years [IQR 0–7] vs. 0.2 years [IQR 0–4], P = 0.10). The median number of cigarettes per day and median years smoking did not differ significantly between cases and controls. There were no significant differences in alcohol consumption between cases and controls; 16% of cases reported heavy drinking (i.e., ≥15 drinks per week) compared to 14% of controls.

Significant differences were found among the three smoking categories with respect to age, CD4 count, years smoking, and alcohol consumption (Table 2). Median CD4 count and median viral load were lowest among individuals who never smoked. Participants reporting current smoking were more likely to report heavy drinking than participants reporting former (25% vs. 11%, P<0.001) or never smoking (25% vs. 9%, P<0.001).

**Table 2:** Socio-demographic characteristics according to smoking history among men with HIV in Soweto, Johannesburg, South Africa (n = 279).

	Current Smoking (n = 92)	Former Smoking (n = 117)	Never Smoking (n = 70)	
Characteristic°	n (%)	n (%)	n (%)	*P*‡
Cases with TB	49 (53)	66 (56)	31 (44)	0.27
Controls without TB	43 (47)	51 (44)	39 (56)	
Age (years), (n = 278)				
Median [IQR]	37 [32–45]	41 [35–49]	36 [32–42]	**<0.001**
Education, (n = 272)				
5th grade or lower	14 (16)	13 (11)	8 (12)	0.21
6th grade to 11th grade	59 (66)	81 (71)	39 (57)	
12th grade or higher	17 (19)	20 (18)	21 (31)	
Employed, (n = 261)	42 (49)	51 (47)	31 (46)	0.95
Unemployed	44 (51)	57 (53)	36 (54)	
Monthly household income, (n = 235)†				
0–999 ZAR	45 (56)	52 (52)	36 (56)	0.35
1000–4999 ZAR	31 (38)	37 (37)	18 (28)	
≥5000 ZAR	5 (6)	11 (11)	10 (16)	
Electricity to heat house, (n = 265)	59 (69)	92 (81)	53 (79)	0.12
No electricity to heat house	26 (31)	21 (19)	14 (21)	
BMI (kg/m2), (n = 239)				
Median [IQR]	22 [20–26]	24 [21–26]	24 [22–27]	0.15
CD4 (cells/mm3), (n = 237)				
Median [IQR]	68 [38–145]	71 [36–143]	57 [7–93]	**0.02**
Viral load (copies/ul, thousands), (n = 242)				
Median [IQR]	128 [35–327]	147 [43–500]	82 [17–439]	0.18
Duration of HIV Infection (days), (n = 208)				
Median [IQR]	39 [22–124]	51 [25–233]	42 [22–166]	0.52
Previous TB (n = 260)	11 (13)	17 (16)	5 (8)	0.30
No previous TB disease	74 (87)	92 (84)	61 (92)	
Cigarettes per day, (n = 168)*				
Median [IQR]	5.0 [3–10]	3.0 [3–5]	0 [0–0]	0.18
Years smoking, (n = 188)*				
Median [IQR]	20 [13–26]	23 [16–30]	0 [0–0]	**0.04**
Pack years, (n = 160)*				
Median [IQR]	5 [3–10]	4 [2–9]	0 [0–0]	0.62
Alcoholic drinks/week, (n = 279)				
0 drinks/week	13 (14)	13 (11)	20 (29)	**<0.001**
1–14 drinks/week	56 (61)	91 (78)	44 (63)	
≥ 15 drinks/week	23 (25)	13 (11)	6 (9)	

°Total number of participants with data available listed in parentheses.

^‡^Pearson's chi-squared (or Fisher's exact) test for categorical variables comparing current vs. former vs. never smoking; Kruskal Wallis test for continuous variables.

^†^Participants were classified as having 0–999 ZAR monthly household income if income was unknown ad unemployed.

*P-value for current smoking versus former smoking.

### Association of smoking and pulmonary TB

Self-report of current smoking within 2 months of the interview was significantly associated with a more than 3-fold increase in the odds of PTB compared to never smoking after adjustment (aOR 3.2, 95%CI: 1.3–7.9, P = 0.01) (Table 3). For participants reporting former smoking, the adjusted odds ratio was increased but not statistically significantly (aOR 1.8, 95%CI: 0.8–4.4, P = 0.18). Education above the fifth grade level was associated with a decrease in odds of smoking history among participants.

**Table 3:** Association of pulmonary TB with smoking history among men with HIV in Soweto (n = 169).

	Controls (n = 74)	Cases(n = 95)	aOR(95% CI)*	*P*
Smoking status				
Current smoking	18	37	**3.2 (1.3–7.9)**	**0.01**
Former smoking	29	38	1.8 (0.8–4.4)	0.18
Never smoking	27	20	Ref	
Age, years	74	95	1.0 (0.9–1.0)	0.67
Education				
5th grade or lower	4	16	Ref	
6th grade - 11th grade	52	65	**0.2 (0.1–0.8)**	**0.02**
12th grade or higher	18	14	**0.2 (0.0–0.8)**	**0.03**
Employment status				
Unemployed	37	55	1.1 (0.4–3.0)	0.83
Employed	37	44	Ref	
Monthly household income				
0–999 ZAR	37	57	Ref	
1000–4999 ZAR	27	29	0.7 (0.3–1.9)	0.48
≥5000 ZAR	10	9	0.8 (0.2–3.2)	0.76
CD4 (cells/mm3, hundreds) ϕ	74	95	1.0 (0.7–1.4)	0.88
Viral load (copies/ul, thousands)ϕ	74	95	**1.0 (1.0–1.0)**	**<0.001**
Duration of HIV Infection (days)ϕ	74	95	1.0 (1.0–1.0)	0.89
History of previous TB				
Previous TB	8	13	1.3 (0.4–3.9)	0.62
No previous TB disease	66	82	Ref	
Alcoholic drinks/week				
0 drinks/week	11	17	Ref	
1–14 drinks/week	53	66	0.7 (0.3–1.9)	0.50
≥ 15 drinks/week	10	12	0.4 (0.1–1.6)	0.19

*Odds ratios are adjusted for all other variables with data in this column.

^ϕ^Numbers in table have been rounded to one decimal place. The odds ratios (95% CI) are: CD4 0.971 (0.665–1.418); viral load 1.003 (1.001–1.005); HIV duration 0.999 (0.999–1.000).

## Discussion

In this case-control study of men with HIV in Johannesburg, the odds of smoking history in patients with PTB compared to those without PTB were tripled in patients reporting current smoking within the past 2 months, while patients reporting former smoking nearly doubled this risk. Our data suggests a link between smoking and TB in a high HIV prevalence setting where both risk factors are common. We likely underestimated smoking prevalence in our study, as patients with TB in South Africa may have stopped smoking at symptom onset or as symptoms worsened and been classified as former smokers [18,20]. Given the frequent interactions with the health system required by patients living with HIV and TB, these men represent a key population where practitioners and programs can implement tobacco control interventions to reduce tobacco-related morbidity and mortality and improve TB and HIV therapeutic outcomes [8].

Previous research has consistently demonstrated that smoking increases the risk of TB approximately two-fold [4,11,21,22]; however, our study is among the first to demonstrate this association in men with HIV. Smoking is also associated with poor TB treatment outcomes and a poorer response to life-saving antiretroviral therapy [8,12,23]. An estimated 20% of all TB-related deaths could be prevented if smoking were eliminated in South Africa [10]. Prior studies have demonstrated that alcohol is independently associated with a three-fold increase in TB risk in populations not infected with HIV [11,24–26], but no such independent association was observed in this study among men with HIV. Previous reports of smoking prevalence of 41–52% among men in Soweto are higher than our findings [9,15,27]; further, we found that only 8% reported current smoking and heavy alcohol consumption, which is lower than prior estimates of 53–55% among South African men [28,29].

Although smoking adversely affects the immune system making people more susceptible to TB disease [4,30], the effect of smoking as a predictor of PTB in men with HIV has not been sufficiently studied [31]. Cell mediated immunity and macrophage function, essential to the host defense against *M*. *tb* infection, are directly impaired by exposure to tobacco smoking [4]. The pulmonary compartment of people that currently and previously smoked is poorly prepared to combat *M*. *tb* infection by a number of mechanisms [30,32]. Exposure to cigarette smoke skews the inflammatory mediator profile of macrophages in the lung, thus influencing their handling and elimination of *M*. *tb*, favoring persistence and/or replication of ingested *M*. *tb*, and increasing risk of infection [32]. Natural killer (NK) cytotoxic activity, T cell function suppression in lungs and blood, impaired mucociliary clearance of particles, excessive alveolar macrophage driven regulatory ability, and inadequate cytokine response to infection have also been posited as mechanisms of impaired host defenses in people that smoke [30,32].

The physical environment where exposure to smoking occurs may increase risk of TB transmission and represent an ideal setting for TB detection and tobacco control. Alcohol is more likely to be consumed by people that smoke [26,33] and previous reports from Soweto suggest that 74% of patients with TB drink regularly for extended durations in neighborhood bars called shebeens [34,35]. Our data shows that participants reporting smoking were more likely to drink, and drink heavily, than those never smoking. Drinking places, especially shebeens, have been shown to pose high TB transmission risk, be frequent sites for meeting casual sexual partners, and have a reputation of being frequented by patrons co-infected with TB and HIV [31,34–37]. Moreover, based on geographic cluster analyses we know that TB case load and shebeens cluster together [35,37]. Clustering is particularly seen in areas where more than 40% of the population is unemployed [35]; 49% of our sample reported being unemployed. The relationship between socio-economic status, smoking, and TB exposure in crowded informal settlement settings, such as Soweto, is further complicated by the environmental challenges of poor ventilation, the increased incidence of smoking, and the increased incidence of TB and HIV in these communities. Thus, the association observed between TB and smoking in individuals living with HIV in Soweto may be explained in part by increased risk of exposure to TB infection among these smokers. However, these mechanisms of environmental exposure and immunological impairment suggest opportunities for implementation of evidence-based interventions to protect and educate individuals, especially as evidence exists of willingness to make behavioral changes among men with HIV in South Africa [38]. The ‘MPOWER’ package recommended by WHO’s Framework Convention on Tobacco Control is an example that includes specific actions to address tobacco use that can be readily incorporated into current TB and HIV practices to improve therapeutic outcomes and reduce the unnecessary burden of death and disease due to smoking [8].

This study has several limitations. Enrollment was restricted to men with HIV not on ART, in part due to the higher prevalence of smoking compared to only 11–13% among women in South Africa [9,11,15]. Due to our recruitment methods including inpatient and outpatient cases and controls, there may be unmeasured differences between cases and controls. However, all participants were from the same community and catchment area where CHBH was the only public sector hospital serving this population at the time. We suggest it is very likely that if controls were to require admission to the hospital for TB then they would have been admitted to CHBH. We relied on self-reported data on tobacco use that could lead to potential misclassification of exposure. However, we assessed smoking using a comprehensive, standardized questionnaire. Strong correlation between self-reported and cotinine confirmed tobacco use has been reported in South Africa [5]. We defined current smoking as smoking within the past 2 months to capture those individuals who may have stopped smoking after TB symptom onset but before being diagnosed [9]. We did not assess drug-sensitivity of PTB among cases, though their TB diagnosis was completed in accordance with South African National TB Program Guidelines [14]. We did not exclude TB disease among controls using microbiological confirmatory testing. Instead we relied on past history and symptom screening, potentially misclassifying controls without PTB thereby overestimating the association between PTB and current smoking. Our inclusion criteria requiring controls to have at least two consecutive prior HIV outpatient clinic visits resulted in a longer median duration of HIV infection in controls compared to cases. The sample size for our multivariable model was reduced due to missing data; if the data are not missing at random, it is possible that the estimates from the full sample would have differed from the estimates presented. Finally, while we adjust for several measures of socio-economic status (SES), it is possible that unknown confounding is present in the analysis due to challenges in adjusting for SES.

Tobacco use remains the leading cause of death worldwide. Our results indicate a significant association between current smoking in the past 2 months and PTB among men with HIV. To prevent TB disease among people living with HIV in South Africa, healthcare workers should actively inquire about, and respond to, smoking in the past year [8]. Previous research has demonstrated the successful implementation of smoking cessation services for people with HIV diagnosed with TB in South Africa [39], and using evidence-based strategies from the WHO-recommended ‘MPOWER’ package [8]. As the epidemiology of TB, HIV, and tobacco collide, it is imperative to address tobacco use as the leading cause of preventable death to ensure that patients with TB and HIV can realize the benefits of life-saving TB treatment and ART [8]. Future research is needed to assess the impact of extensive programs to encourage adults with HIV to stop smoking to reduce the odds of TB and promote general health for men with HIV.
